# *Leptospira* spp. and *Toxoplasma gondii* in stranded representatives of wild cetaceans in the Philippines

**DOI:** 10.1186/s12917-019-2112-5

**Published:** 2019-10-26

**Authors:** Marie Christine M. Obusan, Ren Mark D. Villanueva, Maria Auxilia T. Siringan, Windell L. Rivera, Lemnuel V. Aragones

**Affiliations:** 1 Institute of Biology, College of Science, University of the Philippines, Diliman, Quezon City, 1101 Philippines; 2 Natural Sciences Research Institute, College of Science, University of the Philippines, Diliman, Quezon City, 1101 Philippines; 3 Institute of Environmental Science and Meteorology, College of Science, University of the Philippines, Diliman, Quezon City, 1101 Philippines

**Keywords:** *Leptospira* spp., *Toxoplasma gondii*, Cetaceans, Stranding events, Philippines

## Abstract

**Background:**

The stranding events of cetaceans in the Philippines provide opportunities for gathering biological information and specimens, especially from the pelagic forms. As part of an effort to monitor the health of wild cetaceans, this study detected *Leptospira* spp. and *Toxoplasma gondii*, causative agents of the emerging zoonotic diseases leptospirosis and toxoplasmosis respectively, in their stranded representatives. From October 2016–August 2018, 40 cetaceans (representing 14 species) that stranded nationwide were sampled for brain, cardiac muscle, skeletal muscle, kidney, and blood tissues, urine, and sera. These were subjected to molecular, serological, culture, and histopathological analyses to detect the target pathogens.

**Results:**

*T. gondii* was detected in 20 (71%) of the 28 cetaceans with biological samples subjected to either molecular detection through *RE* gene amplification or IgG antibodies detection through agglutination-based serological assay. On the other hand, *Leptospira* was detected in 18 (64%) of 28 cetaceans with biological samples subjected to bacterial culture, molecular detection through 16S rDNA amplification, or IgM antibodies detection through ELISA-based serological assay.

**Conclusions:**

There is the plausibility of toxoplasmosis and leptospirosis in cetacean populations found in the Philippines, however, acute or chronic phases of infections in sampled stranded individuals cannot be confirmed in the absence of supporting pathological observations and corroborating detection tests. Further studies should look for more evidences of pathogenicity, and explore the specific mechanisms by which pelagic cetacean species become infected by *Leptospira* spp. and *T. gondii*. As there is growing evidence on the role of cetaceans as sentinels of land-sea movement of emerging pathogens and the diseases they cause, any opportunity, such as their stranding events, should be maximized to investigate the health of their populations. Moreover, the role of leptospirosis or toxoplasmosis in these stranding events must be considered.

## Background

The waters of the Philippine archipelago harbor a diverse array of marine mammals. To date, 30 marine mammal species, including 28 cetaceans, the dugong (*Dugong dugon*) and the Asian clawless otter (*Aonyx cinereus*), have been confirmed in the Philippines [1]. Based on limited surveys, opportunistic sightings, and stranding events, most of these species range from very rare to common in the Philippines [2]; and regarded as data deficient, endangered, threatened, and vulnerable to extinction, globally. In general, marine mammals live long, grow slowly, and have low fecundity. These features make them not only prone to over-exploitation and exposed to anthropogenic impacts but also good sentinel species [3, 4] – i.e. indicators of oceans and human health. The utility of these species as sentinels for ocean and human health stems from their physiological similarity with humans and their ability to “sample” or concentrate toxins and pathogens from their habitats [5]. Thus, based on the types of diseases and pathogens found in their wild populations, they can indicate human health risks posed by common water resource use [6–9]. Knowledge of their diseases and pathogens is valuable to understand the impacts of subclinical or overt diseases in their populations, routes of infection in marine ecosystems, and risks to other marine and terrestrial vertebrates [10]. This information is needed to prevent the transmission of zoonotic diseases especially at the human-wildlife interface.

One of these zoonotic diseases is leptospirosis, endemic in most tropical and subtropical regions. Southeast Asia is reported as one of its most significant foci regions, and Philippines (with 4.8 annual incidence per million) is 26th among the 28 countries with highest incidence of the disease in humans [11]. The disease is caused by pathogenic spirochetes of the genus *Leptospira* and is propagated in nature through chronic renal infection of carrier animals [12]. Rodents, pigs, dogs, and cattle serve as *Leptospira* reservoirs but different wild and domestic mammals act as accidental hosts for various serotypes of this pathogen [13–15]. Antibodies against *Leptospira* serovars were also detected in reptiles such as snakes, lizards, and turtles [16]. Although it is well documented and characterized in terrestrial species including humans, less information is available regarding its distribution and impact in marine mammals [17]. Previous studies reported the prevalence of leptospirosis or seropositivity to *Leptospira* spp. in the sirenian Peruvian Amazon manatees (*Trichechus inunguis*) [18] as well as in pinnipeds including harbor seals (*Phoca vitulina*) [19, 20], Northern elephant seals (*Mirounga angustirostris*) [21], California sea lions (*Zalophus californianus*) [22–26, 17], and Chilean South American sea lions (*Otaria byronia*) [27]. Most recently, two serovars – Pomona and Calicola – of *Leptospira interrogans* were detected in serum samples of endangered Caspian seals (*Pusa caspica*) in the Caspian Sea off Northern Iran [28]. Information on the prevalence of *Leptospira* in cetaceans is scarce, with the first isolation of the proposed *L. brihuegai* sp. nov from Southern Right Whale (*Eubalaena australis*) that stranded in Argentina reported by Loffler et al. (2015) [29]. Bik et al., (2016) also reported detecting several bacteria with *Leptospira* sequence types in apparently healthy bottlenose dolphins (*Tursiops truncatus*) in California, although none of these sequence types were close to that of pathogenic *L. interrogans* [30].

Another zoonotic disease, toxoplasmosis, is caused by *Toxoplasma gondii*, a coccidian parasite of mammals with cats as definitive host [31]. Previous knowledge considers *T. gondii* as a land-based parasite, until the importance of its transmission by water [32] was implicated by waterborne outbreaks [33] and reports of infections or prevalence in marine mammals including cetaceans [34–43], fissipeds [44, 45], pinnipeds [46–49, 21, 36], and sirenian [50]. In the Philippines, Obusan et al. (2015) reported the occurrence of *T. gondii* in cetacean species [51]. This body of evidence suggests waterborne aspects of toxoplasmosis as a zoonotic disease as well as the utility of marine mammals to serve as surrogates for studying its emergence in the marine environment [36].

The stranding events of cetaceans in the Philippines provide opportunities for gathering biological information and specimens, especially from the pelagic forms. Based on Aragones et al., (2017), the trend in the frequency of local marine mammal stranding events in the Philippines has been increasing through the years, with a total of 713 strandings from 2005-August 2016 and an annual average of 65 events. These strandings are most likely to be responded in the so-called regional hotspots, administrative regions with highest stranding frequencies. As an archipelago, the Philippines is divided into 17 regions for administrative purposes, and Regions I, II, III, V, and VII, are the marine mammal stranding hotspots [52]. Cetacean stranding events have been associated with infection by pathogenic agents occurring during or after periods of immune suppression [53, 54]. However, proving this, as well as identifying the specific cause of a stranding event is a difficult task, as there is usually a synergy of factors that may cause an animal to strand. While the presence of pathogens (and the diseases associated with them) does not necessarily explain the causation of a stranding event, it indicates the health status of wild cetacean populations as well as the conditions of their habitats. As part of an effort to monitor the health of cetaceans found in the Philippines, this study detected *Leptospira* spp. and *T. gondii* in different biological samples obtained from individuals that stranded in the country from October 2016–August 2018.

## Results

### Stranded cetaceans

**Fig. 1:**
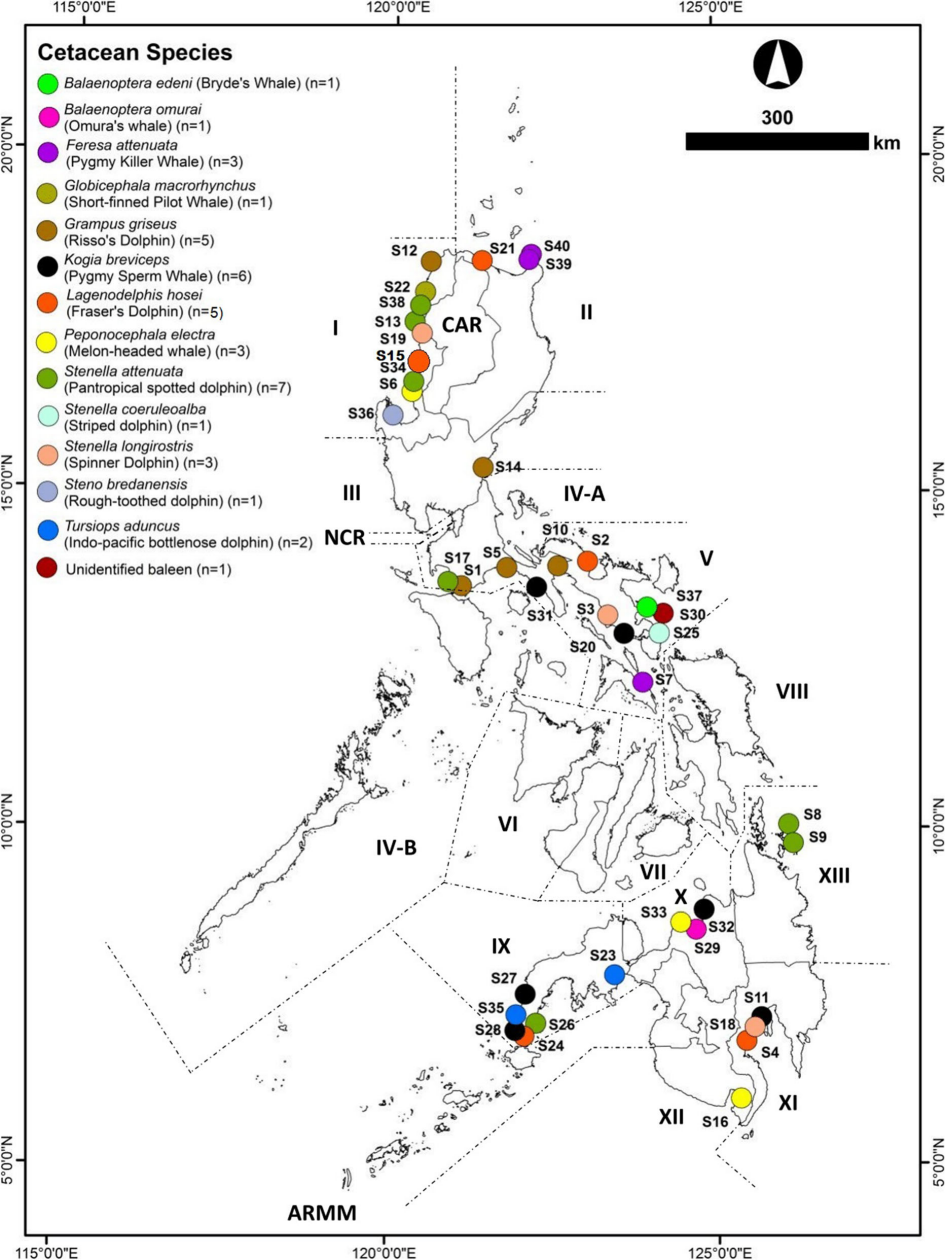
Cetacean stranding sites. Forty individuals confirmed to belong to 13 cetacean species that stranded in Philippine waters from October 2016 to August 2018, were sampled for biological materials

Forty (40) cetaceans that stranded in Philippine waters from October 2016 to August 2018, were sampled for biological materials (Table 1). Thirty-seven (37) of these were involved in single stranding events. Three (3) cetaceans were from mass stranding events; two of which were sampled from one event while one came from a separate event. Stranded individuals represented 14 cetacean species (Fig. 1). The majority of these individuals were alive when they stranded (*n* = 26); 21 of them died while being responded or rehabilitated while three were released back into the wild.

**Table 1:** Stranded cetaceans that were sampled from October 2016–August 2018

Strander No.	Common name	Sex	Age Class	Physical Preservation Code	Type of Stranding	Date of Stranding	Season of Stranding	Region of Stranding
S1	*Grampus griseus* (Risso’s dolphin)	Female	Adult	2	Single	19 October 2016	Lull before NE	Region IV-A
S2	*Lagenodelphis hosei* (Fraser’s dolphin)	Male	Adult	2	Single	27 February 2017	NE	Region V
S3	*Stenella longirostris* (spinner dolphin)	Female	Adult	2	Single	04 March 2017	NE	Region V
S4	*Lagenodelphis hosei* (Fraser’s dolphin)	Female	Adult	2	Single	09 March 2017	NE	Region XI
S5	*Grampus griseus* (Risso’s dolphin)	Unknown	Subadult	2	Single	29 March 2017	NE	Region IV-A
S6	*Peponecaphala electra* (melon-headed whale)	Female	Unknown	2	Single	30 April 2017	Lull before SW	Region I
S7	*Feresa attenuata* (pygmy killer whale)	Unknown	Adult	2	Mass	02 May 2017	Lull before SW	Region V
S8	*Stenella attenuata* (Pantropical spotted dolphin)	Female	Adult	2	Single	07 May 2017, 0800H	Lull before SW	Region XIII
S9	*Stenella attenuata* (Pantropical spotted dolphin)	Male	Adult	2	Single	07 May 2017, 1400H	Lull before SW	Region XIII
S10	*Grampus griseus* (Risso’s dolphin)	Unknown	Adult	2	Single	09 May 2017	Lull before SW	Region V
S11	*Kogia breviceps* (pygmy sperm whale)	Male	Adult	2	Single	16 May 2017	Lull before SW	Region XI
S12	*Grampus griseus* (Risso’s dolphin)	Female	Neonate	1	Single	15 June 2017	SW	Region I
S13	*Stenella attenuata* (Pantropical spotted dolphin)	Female	Subadult	2	Single	21 June 2017	SW	Region I
S14	*Grampus griseus* (Risso’s dolphin)	Unknown	Adult	2	Single	23 June 2017	SW	Region III
S15	*Lagenodelphis hosei* (Fraser’s dolphin)	Unknown	Unknown	1	Single	02 July 2017	SW	Region I
S16	*Peponocephala electra* (melon-headed whale)	Male	Adult	2	Single	03 July 2017	SW	Region XII
S17	*Stenella attenuata* (Pantropical spotted dolphin)	Female	Subadult	1	Single	28 July 2017	SW	Region IV-A
S18	*Stenella longirostris* (spinner dolphin)	Female	Subadult	2	Single	31 August 2017	SW	Region XI
S19	*Stenella longirostris* (spinner dolphin)	Female	Subadult	2	Single	30 September 2017	SW	Region I
S20	*Kogia breviceps* (pygmy sperm whale)	Female	Adult	2	Single	09 November 2017	NE	Region V
S21	*Lagenodelphis hosei* (Fraser’s dolphin)	Female	Adult	2	Single	01 December 2017	NE	Region II
S22	*Globicephala macrorhynchus* (short-finned pilot whale)	Female	Adult	2	Single	05 December 2017	NE	Region I
S23	*Tursiops aduncus* (Indo-Pacific bottlenose dolphin)	Female	Adult	1	Single	15 January 2018	NE	Region IX
S24	*Stenella attenuata* (Pantropical spotted dolphin)	Male	Adult	2	Single	16 January 2018	NE	Region IX
S25	*Stenella coeruleoalba* (striped dolphin)	Female	Adult	1	Single	15 February 2018	NE	Region V
S26	*Lagenodelphis hosei* (Fraser’s dolphin)	Female	Adult	2	Single	17 April 2018	Lull before SW	Region IX
S27	*Kogia breviceps* (pygmy sperm whale)	Male	Subadult	2	Single	26 April 2018	Lull before SW	Region IX
S28	*Kogia breviceps* (pygmy sperm whale)	Female	Adult	2	Single	27 April 2018	Lull before SW	Region IX
S29	*Balaenoptera omurai* (Omura’s whale)	Female	Neonate	2	Single	30 April 2018	Lull before SW	Region X
S30	*Balaenoptera* sp. (unidentified baleen)	Unknown	Adult	2	Single	03 May 2018	Lull before SW	Region V
S31	*Kogia breviceps* (pygmy sperm whale)	Male	Adult	2	Single	06 May 2018	Lull before SW	Region IV-A
S32	*Kogia breviceps* (pygmy sperm whale)	Female	Adult	2	Single	10 May 2018	Lull before SW	Region X
S33	*Peponocephala electra* (melon-headed whale)	Female	Adult	2	Single	17 May 2018	Lull before SW	Region X
S34	*Stenella attenuata* (Pantropical spotted dolphin)	Unknown	Subadult	2	Single	25 May 2018	Lull before SW	Region I
S35	*Tursiops aduncus* (Indo-Pacific bottlenose dolphin)	Male	Adult	2	Single	28 May 2018	Lull before SW	Region IX
S36	*Steno bredanensis* (rough-toothed dolphin)	Female	Adult	1	Single	02 July 2018	SW	Region I
S37	*Balaenoptera edeni* (Bryde’s whale)	Unknown	Neonate	1	Single	03 July 2018	SW	Region V
S38	*Stenella attenuata* (Pantropical spotted dolphin)	Male	Adult	1	Single	08 August 2018	SW	Region I
S39	*Feresa attenuata* (pygmy killer whale)	Male	Adult	1	Mass	17 August 2018	SW	Region II
S40	*Feresa attenuata* (pygmy killer whale)	Female	Adult	1	Mass	17 August 2018	SW	Region II

Stranding events were recorded in Luzon (*n* = 25) and Mindanao (*n* = 15) Islands (Fig. 1). More than half (*n* = 21) of these strandings occurred in administrative regions included in the top five stranding hotspots: Region I (*n* = 9), Region II (*n* = 3), Region III (*n* = 1), and Region V (*n* = 8). The rest occurred in Region IV-A (*n* = 4), Region X (*n* = 3), Region IX (*n* = 6), Region XI (*n* = 2), Region XII (*n* = 2), and Region XIII (*n* = 2). Most of the strandings (*n* = 16) occurred during the lull period before southwest (SW) monsoon, while only one was recorded during the lull before northeast (NE) monsoon. The rest occurred during SW monsoon (*n* = 13) and NE monsoon (*n* = 10).

The following biological samples were obtained (with *n* = number of cetacean individuals): brain tissues (*n* = 10), cardiac muscle tissues (*n* = 14), and skeletal muscle tissues (15) were used for molecular detection of *T. gondii* while kidney tissues (*n* = 12) were used for molecular detection, histopathological examination, and isolation of *Leptospira* spp. Bacterial isolation was also done using urine (*n* = 2) and blood samples (*n* = 22). Moreover, all blood samples were subjected to molecular detection of both target pathogens. Serum samples (*n* = 7) were used to detect *T. gondii* IgG antibodies and *Leptospira* IgM antibodies. The number of detection methods to which each cetacean was subjected depended on the type of biological sample/s collected considering the physical preservation and condition of the animal.

### *T. gondii* detection

For the detection of *T. gondii*, 15 individuals (S1, S2, S3, S4, S5, S10, S11, S12, S13, S16, S18, S21, S22, S24, and S25) had tissue/s positive for the target *RE* gene and six (S15, S24, S36, S37, S39, and S40) were seropositive for IgG antibodies against the protozoan parasite. One individual (S24) was both sero- and *RE* gene- positive. Another individual (S25) was *RE-gene* positive but sero-negative. Among 28 cetaceans with biological samples subjected to either gene-specific PCR assay or agglutination-based serological assay, *T. gondii* was detected in 20 (71%) individuals (Table 2).

**Table 2:** Summary of results for detection of target pathogens

Strander Code	Cetacean Species (and common name)	*T. gondii* detection by PCR				*T. gondii* detection by LAT	*Leptospira* detection by PCR		*Leptospira* culture	*Leptospira* detection by ELISA
							Blood	Kidney		
		Blood	Cardiac	Skeletal	Brain					
S1	*Grampus griseus* (Risso’s dolphin)	–	*	+	*	*	–	*	–	*
S2	*Lagenodelphis hosei* (Fraser’s dolphin)	–	*	+	+	*	+	–	+^C^	*
S3	*Stenella longirostris* (spinner dolphin)	–	+	+	+	*	+	–	+^A^	*
S4	*Lagenodelphis hosei* (Fraser’s dolphin)	+	+	+	+	*	+	–	+^A^	*
S5	*Grampus griseus* (Risso’s dolphin)	–	+	–	*	*	–	–	*	*
S6	*Peponecaphala electra* (melon-headed whale)	*	*	*	*	*	*	*	+^B^	*
S7	*Feresa attenuata* (pygmy killer whale)	*	*	*	*	*	*	*	*	*
S8	*Stenella attenuata* (Pantropical spotted dolphin)	*	*	*	*	*	*	*	*	*
S9	*Stenella attenuata* (Pantropical spotted dolphin)	*	*	*	*	*	*	*	*	*
S10	*Grampus griseus* (Risso’s dolphin)	+	+	+	+	*	+	–	–	*
S11	*Kogia breviceps* (pygmy sperm whale)	*	+	+	+	*	*	*	*	*
S12	*Grampus griseus* (Risso’s dolphin)	+	*	*	*	*	–	*	–	*
S13	*Stenella attenuata* (Pantropical spotted dolphin)	*	+	+	*	*	*	*	+^B^	*
S14	*Grampus griseus* (Risso’s dolphin)	*	–	*	*	*	*	*	+^A^	*
S15	*Lagenodelphis hosei* (Fraser’s dolphin)	–	*	*	*	+	+	*	–	+
S16	*Peponocephala electra* (melon-headed whale)	+	+	*	+	*	+	–	+^D^	*
S17	*Stenella attenuata* (Pantropical spotted dolphin)	*	*	*	*	*	*	*	*	*
S18	*Stenella longirostris* (spinner dolphin)	+	–	+	+	*	–	–	–	*
S19	*Stenella longirostris* (spinner dolphin)	–	*	*	*	*	+	*	–	*
S20	*Kogia breviceps* (pygmy sperm whale)	–	–	*	–	*	+	–	–	*
S21	*Lagenodelphis hosei* (Fraser’s dolphin)	–	+	+	*	*	–	–	+^A^	*
S22	*Globicephala macrorhynchus* (short-finned pilot whale)	+	+	–	+	*	+	*	*	*
S23	*Tursiops aduncus* (Indo-Pacific bottlenose dolphin)	*	*	*	*	*	*	*	*	*
S24	*Stenella attenuata* (Pantropical spotted dolphin)	+	+	+	*	+	–	*	–	+
S25	*Stenella coeruleoalba* (Striped dolphin)	+	*	*	*	–	–	*	–	+
S26	*Lagenodelphis hosei* (Fraser’s dolphin)	*	*	*	*	*	*	*	*	*
S27	*Kogia breviceps* (pygmy sperm whale)	–	–	–	*	*	–	–	–	*
S28	*Kogia breviceps* (pygmy sperm whale)	*	*	*	*	*	*	*	*	*
S29	*Balaenoptera omurai* (Omura’s whale)	*	*	*	*	*	*	*	*	*
S30	*Balaenoptera* sp. (unidentified baleen)	*	*	*	*	*	*	*	*	*
S31	*Kogia breviceps* (pygmy sperm whale)	–	*	–	–	*	–	–	–	*
S32	*Kogia breviceps* (pygmy sperm whale)	*	*	*	*	*	*	*	*	*
S33	*Peponocephala electra* (melon-headed whale)	–	*	*	*	*	–	*	–	*
S34	*Stenella attenuata* (Pantropical spotted dolphin)	–	*	–	*	*	–	–	–	*
S35	*Tursiops aduncus* (Indo-Pacific bottlenose dolphin)	–	*	*	*	*	–	*	–	*
S36	*Steno bredanensis* (rough-toothed dolphin)	*	*	*	*	+	*	*	*	+
S37	*Balaenoptera edeni* (Bryde’s whale)	–	*	*	*	+	–	*	–	+
S38	*Stenella attenuata* (Pantropical Spotted dolphin)	*	*	*	*	*	*	*	*	*
S39	*Feresa attenuata* (pygmy killer whale)	*	*	*	*	+	*	*	*	+
S40	*Feresa attenuata* (pygmy killer whale)	*	*	*	*	+	*	*	*	+
Total positive results out of screened cetaceans		8/22	10/14	10/15	8/10	6/7	9/22	0/12	15/23	7/7

+ positive for *T. gondii* or *Leptospira* spp.

- negative for *T. gondii* or *Leptospira* spp.

+^A^ one putative leptospire isolate

+^B^ two putative leptospire isolates

+^C^ three putative leptospire isolates

+^D^ four putative leptospire isolates

*biological sample for testing not available/enough

### *Leptospira* spp. detection

**Fig. 2:**
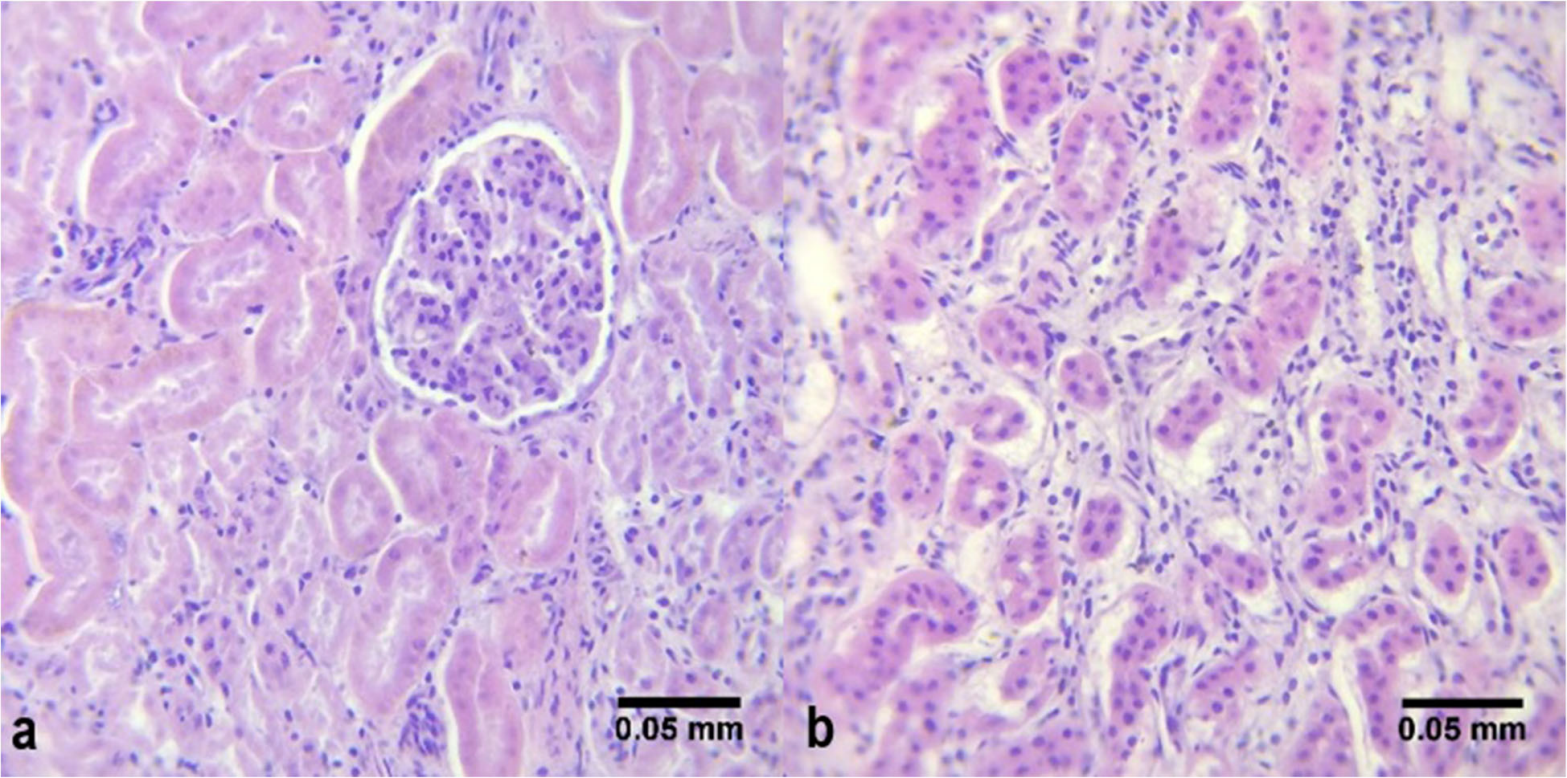
Interstitial nephritis in the kidney of a melon-headed whale (S6). The kidney tissue exhibited leptospirosis-associated tubulointerstitial nephritis, characterized by mild-thickening of basement membrane capillaries and necrosis of convoluted tubular epithelium

**Fig. 3:**
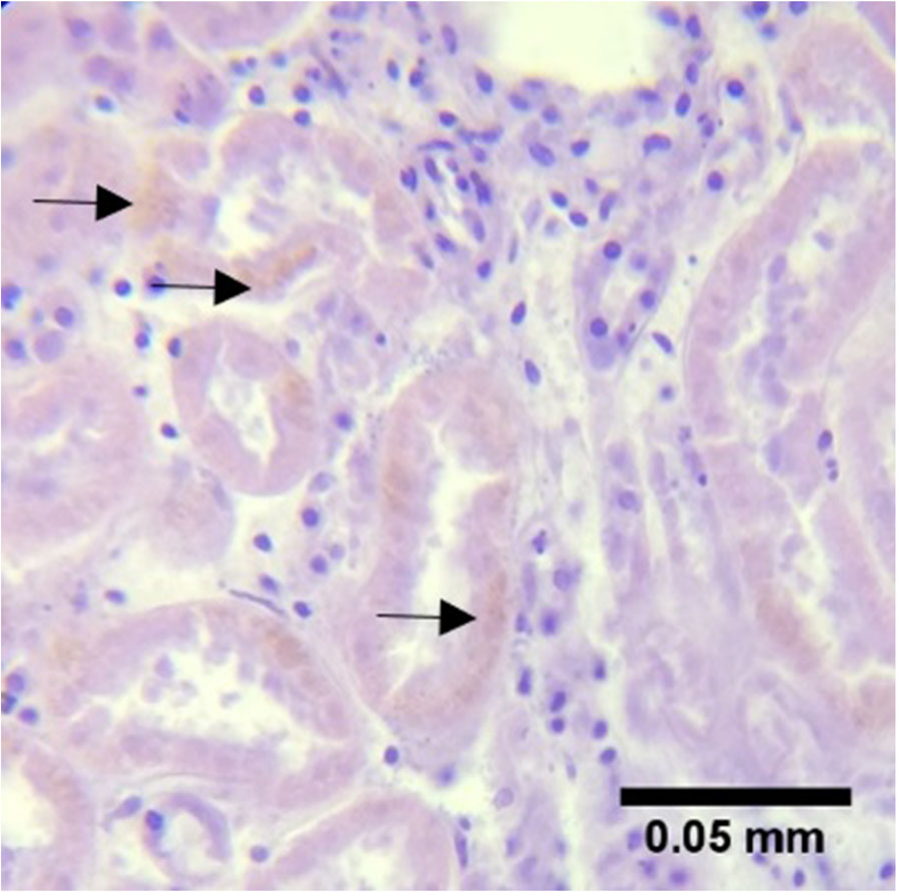
Hemosiderosis: brown granular pigments (black arrows). Hemosiderosis was observed in the kidney tissue of a melon-headed whale (S6), characterized by the presence of hemosiderin pigments from hemoglobin degradation

*Leptospira* was detected in the blood samples of nine individuals (S2, S3, S4, S10, S15, S16, S19, S20, and S22) through 16S rDNA amplification. This detection represents both pathogenic and non-pathogenic species of the genus as targeted by the primers used. Seven individuals (S15, S24, S25, S36, S37, S39, and S40) were sero-positive for *Leptospira* IgM antibodies. Two (2) were successfully sequenced from 15 putative leptospires that were isolated: isolate 4KT1.2 (from the kidney of S4) and isolate 6KT1.2 (from the kidney of S6) has 98 and 99% sequence similarity respectively to *L. interrogans* serovar Copenhageni strain FDAARGOS_203 (NCBI Accession No. CP020414). S6 exhibited leptospirosis-associated tubulointerstitial nephritis (Fig. 2), characterized by mild thickening of basement membrane capillaries and necrosis of convoluted tubular epithelium [22]. As this lesion was observed concurrent to bacterial isolation, it is likely that this cetacean individual had recent *Leptospira* infection. In addition, hemosiderosis was observed (Fig. 3). Both the isolates were found to tolerate different seawater concentrations (1, 3, 5, 7 and 10%) up to 2 days of incubation when grown in EMJH Media and Korthof Media, indicating their ability to survive in the marine environment. Out of 28 cetaceans with biological samples subjected to any of the detection methods (culture, gene-specific PCR assay, or ELISA-based serological assay), 18 (64%) individuals were positive for *Leptospira* spp. (Table 2).

## Discussion

The detection of potentially pathogenic *Leptospira* spp. in cetaceans underscores the need to understand how this bacterial group moves through hosts and environments that are not usually identified in its cycle of transmission. Prager et al. (2013) reported the asymptomatic carriage of *Leptospira* in both wild and captive sea lions, giving clues on the long-term circulation of leptospirosis in their habitats [26]. Leptospirosis was also significantly associated with close proximity to dog parks as well as high dog-park density in California sea lions [62]. However, such case represents a coastal environment that directly receives land-based effluents. How species of *Leptospira* become transmitted to pelagic cetacean species (e.g., in this study, Fraser’s dolphin (*L. hosei)*, melon-headed whale (*P. electra*), and others) that stay in the open sea remains to be understood in relation to the ability of these bacterial group to remain viable in saltwater conditions. Elsewhere, reports on leptospirosis and seroprevalence to *Leptospira* were mostly on pinniped species and involved cases prompted by epizootics [24]. Among cetaceans, *Leptospira* spp. were only reported in Southern Right Whale (*E. australis*) in Argentina [29] and in bottlenose dolphins (*T. truncatus*) in California [30].

While leptospirosis in marine mammals is not yet substantially characterized, interpretations of detection methods may use as reference, the descriptions in humans and other mammals. Leptospirosis in humans has two phases: (1) acute phase, which is usually the first 7 days of illness (may end 3–7 days), when leptospires can be cultured and detected from the blood; and (2) immune phase, which can last for 4–30 days, when antibodies can be detected in the blood and leptospires can be cultured from the urine [63]. The limitations of serology include (1) lack of antibodies at the acute phase; (2) “anamnestic response” or the rise in antibody titer that is directed against a previous infecting serovar; (3) high degree of cross-reactions between serogroups especially during the acute phase; and (4) differences in the sensitivity of tests, for example, earlier detection by ELISA from day 6–8 (which may cover the acute phase) compared with MAT [64]. In the case of the nine stranders (S2, S3, S4, S10, S15, S16, S19, S20, and S22) that were positive in blood PCR detection, the presence of acute infection cannot be confirmed since 16S rDNA targeted both pathogenic and non-pathogenic *Leptospira* spp. Included in these stranders are S15 and S4, which had IgM in the blood and bacterium isolated from the kidney respectively. It is possible that a non-pathogenic *Leptospira* species was detected by PCR in the blood of these individuals. If this is the case, then the detected IgM in S15 was against a pathogenic serovar, or if indeed such serovar was amplified in the blood, then the IgM might have been detected early in the acute phase given the reported early detection by ELISA. As the kidney isolates from S4 and S6 were most phylogenetically related to *L. interrogans*, these cetaceans may have chronic renal carriage of leptospires (immune phase of infection) or active infection if presented with clinical symptoms such as in the case of dogs [65]. The seven stranders (S15, S24, S25, S36, S37, S39 and S40) that were sero-positive for IgM might be in the immune phase of leptospirosis. The presence of the anti-leptospiral IgM may be attributed to the persistence of the antibody after infection, frequent reinfection with leptospires in endemic areas, or cross-reaction with other infectious agents [66]. Overall, there is evidence for the exposure of sampled cetaceans to pathogenic *Leptospira* spp., but it is rather difficult to confirm the phase of infection given the limitations in the detection tests and biological samples.

It must be noted that there is 100% sero-positivity in all sera qualified for the detection of IgM antibodies against *Leptospira*. However, this result is limited by the fact that SERION *Leptospira* IgM-ELISA was only evaluated to detect the complexes formed by human IgM and *Leptospira* antigens representing known serovars bound to goat antihuman IgM. The test was used as an accepted surrogate to the gold standard but laborious and time-consuming Microscopic Agglutination Test (MAT) which requires the maintenance of live serovars [63]. The test’s protocol claims the likelihood of cross-reactivity of goat antihuman IgM with IgM from other species [63]. While specific information on cross-reactivity of cetacean and human antibodies to *Leptospira* antigens are yet to be available, it is said that humans follow the classical IgM response to *Leptospira* antigens similar to animals [63]. The suitability of cetaceans as sentinels for marine zoonoses such as leptospirosis may be supported by evidences of cross-reactivity of (1) antibodies to human antigens and tissues of the bottlenose dolphin (*T. truncatus*) [67] suggesting that the applied serological assay has a reasonable sensitivity at least for many cetacean species; (2) human and bovine antibodies in paraffin-wax embedded tissues of striped dolphin (*S. coeruleoalba*) [63]; and (3) commercially available terrestrial-specific antibodies (from pig, rat, mice, and humans) to dolphins, allowing the characterization of the immune cell subsets of under human care and free-ranging dolphins [63].

In the Philippines, the detection of pathogenic *Leptospira* spp. in coastal soil after the storm surge brought about by typhoon Haiyan that devastated the Eastern Visayas part of the Philippines was reported by Saito et al. (2014) [63]. Their report confirmed the survival of pathogenic *Leptospira* sp. in seawater for 4 d, showing the ability of soil-inhabiting leptospires to persist even after a storm surge, and thus, the likelihood of a leptospirosis outbreak during seawater inundation episodes brought about by natural disasters. Khairani-Bejo (2004) reported the short survival of an isolate identified as *L. interrogans* serovar Hardjo in a medium with 3.78 and 3.85% salt content and pH of 6.5 to 6.8 [63]. The novel *Leptospira* spp. strain Manara isolated from Southern Right Whale tolerated at least 5% seawater in medium for 48 h [29]. Likewise, the present study supports the survival of *Leptospira* spp. survival in seawater as the two isolates from stranded cetaceans were found to tolerate up to 10% seawater in media for 2 d. Seawater-tolerant leptospires may gain entry in cetaceans through direct contact with infected urine of infected or reservoir animals, or exposure to soil, water, and food that have been contaminated with infected urine. Among different hosts, transmissions through bites, tissue ingestion, sexual contact, breast milk, and placenta were also reported [63].

The known symptom of leptospirosis in marine mammals is interstitial nephritis, which is presented with clinical signs of impaired renal function including dehydration, polydipsia or excessive thirst, muscular tremors, abdominal pain, vomiting, and depression [24, 22]. The renal lesions in the melon-headed whale (*P. electra*) were consistent with those associated with leptospirosis in California sea lions (*Z. californianus*) and Northern elephant seals (*M. angustirostris*) that stranded along the coast of California [21–23]. The hemosiderosis observed in this particular cetacean, characterized by the presence of hemosiderin pigments from hemoglobin degradation, may result from infection, dietary deficiencies, excessive dietary iron (which increases susceptibility to bacterial infections and organ dysfunction), corticosteroids, and other toxins [63]. As bacterial infection can cause hemosiderosis, there is reason to suspect that this, together with tubulointerstitial nephritis, resulted from the infection of *Leptospira* sp. isolated from the kidney of the cetacean.

*T. gondii* in cetaceans found in the Philippines was first reported by Obusan et al. (2015) [51]. Since then, the protozoan parasite has been included as one of the target pathogens for the screening of cetaceans that strand in the country. As it is in the first study, the cetacean species where *T. gondii* was detected in this study were also pelagic such as the Risso’s dolphin (*G. griseus*), Fraser’s dolphin (*L. hosei)*, spinner dolphin (*S. longirostris*), and others. This pose an interesting question as to how these cetaceans become exposed or infected with the parasite. The prevalence of toxoplasmosis in their populations is possible, given that *T. gondii* was detected in tissues of stranders through PCR and serological assays. However, the present interpretations are limited by the fact that only *T. gondii* specific IgG was detected, and that the presence of this type of antibody alone cannot unequivocally indicate a chronic infection. Recent reports suggest *T. gondii* specific IgM and/or IgG fail/s to differentiate between acute (3–6 months) and chronic (beyond 6 months) phases of toxoplasmosis as they are detected in both phases (80). In the case of humans, it is suggested that diagnosis for toxoplasmosis must be interpreted based on a combination of serological and molecular detection methods. For example, an acute infection can be indicated by concurrent IgM and low IgG avidity or a chronic infection can be indicated by concurrent IgM and high IgG avidity or IgG and high IgG avidity [63]. In addition, the use of molecular detection such as gene-specific PCR is helpful for confirming disseminated infection due to the systemic nature of toxoplasmosis as well as propagation of infection through body fluids [84; 81]. There is one cetacean (S24) that was positive for both IgG in the serum and *RE* gene in the blood and cardiac and skeletal muscles, indicating a disseminated infection. Another individual (S25) was *RE-gene* positive but sero-negative; in this case, there is the likelihood that IgM antibodies were present but were not detected given the limitations of testing kits [63]. The other 15 cetaceans that were positive in PCR might either have disseminated infection (i.e., positive detection in blood of cetacean stranders (S4, S10, S12, S16, S18, S22, S24, and S25), and in both blood and muscles of cetacean stranders (S1, S2, S3, S4, S5, S10, S11, S12, S13, S16, S18, S21, S22, S24, and S25) or latent infection (i.e., positive detection of tissue cysts only in muscles of cetacean stranders S1, S2, S3, S5, S11, S13, and S21). The other five sero-positive cetaceans (S15, S36, S37, S39, and S40) that are negative in PCR detection can be safely said to have been exposed to the parasite.

Infection by *T. gondii* can occur transplacentally, or through the ingestion of food or water contaminated by oocysts as well as consumption of tissue with the bradyzoite stage of the parasite [44]. It is interesting to note that dolphins drink very small amount of water [﻿44﻿] and cetaceans in general are known to consume cephalopods, shrimps, and fishes [63] poikilothermic preys that are not hosts to *T. gondii*. However, Massie et al. (2010) proved that northern anchovies (*Engraulis mordax)* and Pacific sardines (*Sardinops sagax*) serve as biotic vectors for *T. gondii* transmission in marine environment [63]. With the elimination of carnivory feeding as the possible source of *T. gondii* in cetaceans, it is likely that oocyst contamination of marine water and prey item is a risk factor for infection, thus supporting pollution of their habitat by land to sea movement of the parasite. Such contamination is said to be coming from effluents as well as ship runoff waters containing oocysts [8, 37], which can survive in the environment for years (Black and Boothroyd, 2000). Di Guardo and Mazzariol (2013) asserted that direct oocyst contamination of seawater from land-based effluents may explain the infection of coastal species such as bottlenose dolphins, however, in the case of *T. gondii* detection in pelagic species, the possibility of an “open sea *T. gondii* life cycle” that is different from the known land and benthic protozoan cycles must be considered [63]. It is also possible that transmission of the parasite or infection happened during migration. For example, toxoplasmosis is known to affect striped dolphins (*S. coeruleoalba*) in the Mediterranean region [37]. A stranded striped dolphin was also one of the samples in this study, and *T. gondii* was amplified from its blood. Striped dolphins are widely-distributed worldwide; they are found in warm temperate and tropical waters of Atlantic, Indian and Pacific Ocean [63]. Cetaceans are known to migrate, but information is lacking regarding the migration patterns and abundance ranges of many cetacean species in the Philippines.

Screening stranded cetaceans for the presence of target pathogens may help explain the possible cause/s of their stranding events and guide decisions in cases of medical intervention or rehabilitation. For example, the melon headed whale or *P. electra* (S6) may have stranded due to leptospirosis evidenced by tubulointerstitial nephritis with concurrent isolation of *Leptospira* sp. However, predation may have also contributed to the debility of the animal as shark bite was seen in its body. The dolphin was rehabilitated but died on 22 May 2017 (more information on this strander can be accessed through http://newsinfo.inquirer.net/895271/whale-nursed-back-to-health-in-la-union) [63]. Another strander, the rough-toothed dolphin or *S. bredanensis* (S36) was found to be seropositive for *T. gondii* and *Leptospira* spp., confirming exposure to the pathogens. For its rehabilitation, the dolphin was first brought to a fish tank in BFAR-RMaTDeC (Regional Mariculture Technodermo Center) in Lucap Wharf, Alaminos, and then to the sea pen in Cariaz Island of Hundred Islands National Park, Pangasinan, Philippines. During the early days of rehabilitation, the animal showed symptoms of health problems which include diarrhea and expulsion of placental-like tissues indicative of either recent calf delivery or abortion prior to the stranding event. The dolphin was also observed to have abnormally short respiratory intervals, followed by straining and flexing, which could be an effort to expel placenta. Thus, antibiotics, pain relievers, oxytocin, dinoprost, and calcium were given to ease the symptoms and facilitate expulsion of any remaining placental tissues (L.J. Suarez, pers. comm., July 2018). While toxoplasmosis and leptospirosis are reported to cause abortion in animals [63], the limited serology cannot conclusively support such in the absence of corroborating findings due to limitations in the collected biological samples (e.g., available for PCR assay and histopathological analyses). The dolphin had IgG antibodies against *T. gondi,* which cannot differentiate between acute or chronic toxoplasmosis, and had IgM antibodies against *Leptospira*, which more likely indicate immune rather than acute phase of leptospirosis. As time progressed, continuous improvement in the dolphin’s health and physical condition was observed until it was successfully released back into the wild on 21 August 2018 at Lingayen Gulf (news story on this strander can be accessed through http://www.pna.gov.ph/articles/1045798) [63]. Considering the foregoing cases, active infections cannot be confirmed in the absence of supporting pathological observations and detection tests.

## Conclusions

*Leptospira* spp. and *T. gondii* were detected in cetaceans that stranded in the Philippines from October 2016–August 2018. This confirmed the plausibility of leptospirosis and toxoplasmosis in their populations, and the possible role of these infections in their local stranding events. Further studies should explore the specific mechanisms by which pelagic cetacean species become infected by *Leptospira* spp. and *T. gondii*, as well as the routes of transmission of these microorganisms in the marine environment. As there is growing evidence on the role of cetaceans as sentinels of land-sea movement of emerging pathogens and the diseases they cause, any opportunity, such as their stranding events, should be maximized to investigate the health of their populations through their stranded representatives. Moreover, experiences in sampling and rehabilitating stranded cetaceans should guide future practices to prevent zoonotic transmissions at the human-animal interface.

## Methods

### Stranded cetaceans

Cetacean stranding events that occurred in the Philippines from October 2016–August 2018 were monitored and responded through collaboration with Philippine Marine Mammal Stranding Network (PMMSN) as well as Department of Agriculture’s Bureau of Fisheries and Aquatic Resources (DA-BFAR). PMMSN has 12 regional and 32 provincial chapters that have marine mammal stranding response teams mandated by BFAR regional offices. The members and volunteers of the teams report any stranding event and the communication is coursed through channels until the information is relayed to the research team. Whenever logistically possible, stranding sites were reached by the researchers through land, air, or water travel. In cases wherein the stranding site was very remote and could not be reached immediately, biological material collection proceeded in coordination with PMMSN members who trained on medical aspects of marine mammal stranding response. All provincial chapters of DA-BFAR in different administrative regions have at least one veterinarian who completed such an intensive training course.

Stranded cetacean individuals were characterized in terms of: (1) species; (2) sex (based on genital and/or mammary slits); (3) age class (inferred from length relative to the species); (4) disposition (dead or alive); (5) stranding type (single or mass); (6) stranding site; and (7) stranding season (based on the scheme provided by Wang, 2006) [55]. Biological material collection was done based on the Code system established by the Smithsonian Institution’s Marine Mammal Events Program [56]: Code 1- live animal; Code 2 – fresh (carcass in good condition); Code 3- fair (decomposed, but organs basically intact); Code 4- poor (advanced decomposition); and Code 5 – mummified or skeletal remains.

### Biological materials

Blood was extracted either from the fluke vasculature (Code 1 specimen) or vena cava (Code 2 specimen). For serum recovery, whole blood was placed in serum separator tubes or kept warm until clotted for 30 min and centrifuged at 280 x g for 7 min. Sera were stored at 4 °C - 8 °C and processed within 48 h or stored in a − 80 °C freezer for further analysis. Tissue samples (< 1 cm^3^ each) from kidney (Codes 2–4 specimens), brain (Code 2 specimen only), heart, and skeletal muscles (Codes 2–3 specimens) were obtained by performing necropsy. Urine samples (< 3 mL) were collected from Codes 1–3 specimens. Following necropsy procedure, urine was aspirated from the exposed bladder with a syringe or squeezed through the penis of male individuals [57]. All biological samples were placed in sterile plastic bags, stored at 4 °C while on field work, and transferred immediately (preferably < 12 h) to a − 80 °C freezer for analyses within 6 months.

### Serological assays

Antibodies against *Leptospira* spp. were detected using enzyme-linked immunosorbent assay (SERION ELISA classic *Leptospira* IgM (Institut Virion\Serion GmbH, Warburg, Germany) following manufacturer’s instructions. IgM-ELISA used antigens from *L. biflexa* serovar Patoc strain Patoc I that contains genus specific epitopes for all *Leptospira* serovars. The test was developed to detect the complexes formed by human IgM and Leptospira antigens bound with goat antihuman IgM. The use of this test for non-human hosts relies on cross-reactivity of the goat antihuman IgM with IgM from other mammals. On the other hand, detection of IgG antibodies against *T. gondii* was done using Toxocell Latex Agglutination Test (LAT: BIOKIT Manufacturing Company, Barcelona, Spain), again, following manufacturer’s instructions. The test used a suspension of polystyrene latex particles of uniform size coated with soluble *T. gondii* antigen.

### *Leptospira *culture

*Leptospira* spp. were isolated from blood, urine, and kidney samples using Ellinghausen-McCullough-Johnson- Harris medium (EMJH) following the procedure of Loffler et al. (2015) [29]. Cultures were incubated at 28–30 °C for a maximum of 3 months with dark-field microscopy examination every 15 d to check for turbidity and dinger ring formation as well as characteristic motility of *Leptospira*. Subcultures were prepared in case of positive *Leptospira* spp. growth with simultaneous testing of bacterial survival in halophilic condition through the addition of different seawater concentrations (1, 3, 5, 7 and 10%, v/v) [29].

### Histopathological examination

Kidney tissues were placed in 10% neutral-buffered formalin (with a tissue to fixative ratio of 1:10), embedded in paraffin, and sectioned at 5 μm using a microtome. The tissue sections were then mounted on a slide, and subjected to hematoxylin and eosin staining [58]. Tissue lesions associated with leptospirosis were observed through microscopy.

### Molecular analyses

Extraction of DNA from urine, kidney, blood, brain, and muscle samples proceeded using a commercially available kit (Promega, A1120: Wizard Genomic DNA Purification Kit). Extracted DNA samples were quantified using a spectrophotometer and then polymerase chain reaction (PCR) analyses were performed.

Pathogenic and non-pathogenic *Leptospira* spp. were targeted through nested PCR that amplified 525-bp (first round) and 289-bp (second round) fragments of the 16S rRNA gene [50]. For first amplification, the primers used were: 5′-GGCGGCGCGTCTTAAACATG-3′ and 5′-GTCCGCCTACGCACCCTTTACG-3′ while for second amplification, the primers were 5′ CAAGTCA AGCGGAGTAGCAA-3′ and 5′-CTTAACCTGCTGCCTCCCGTA-3′ [59]. For both amplifications, the thermocycler conditions used were: 94 °C for 5 min, 30 cycles of 60 °C for 2 min, 72 °C for 1.5 min, and 94 °C for 1 min, followed by 60 °C for 2 min and 72 °C for 15 min [59].

The 164 bp region within the 529 bp of the *T. gondii* RE gene was targeted by nested amplifications using primer pairs (1) 5′-TGACTCGGGCCCAGCTGCGT-3′ and 5′-CTCCTCCCTTCGTCCAAGCCTCC-3′; and (2) 5′-AGGGACAGAAGTCGAAGGGG-3′ and 5′-GCAGCCAAGCCGGAAACATC-3′ [60]. The thermocycler conditions used were: (1) for first amplification, 94 °C for 5 min, 30 cycles of 94 °C for 20 s, 55 °C for 20 s, 72 °C for 20 s and 72 °C at 5 min final extension; and (2) for second amplification, 94 °C for 5 min, 35 cycles of 94 °C for 20 s, 55 °C for 20 s, 72 °C for 20 s, and 72 °C at 5 min final extension [61].

Reactions were performed in 25 μl volume with the following concentrations of components: 1X PCR Master Mix (Vivantis: contains *Taq* DNA Polymerase, dNTPs, MgCl2), 1.0 μM assigned primers, 1.5–3.0 μL DNA template, and nuclease-free water adjusted accordingly. Negative controls excluded DNA template. Positive controls included either DNA from *T. gondii* (Su, The University of Tennessee, Knoxville) or reference clinical strain of *L. interrogans* (Rivera, University of the Philippines, Diliman). Electrophoresis of PCR products in TAE (Tris-acetate-EDTA) buffer was performed on agarose gels (2% for 16S rRNA and 1.5% for *flaB* gene) at 8 V/cm with DNA ladder (Vivantis, 100 bp Plus DNA ladder). Following electrophoresis, the gels were stained using GelRed and viewed through UV light exposure. PCR-positive samples were processed for purification, DNA quantification, and sequencing.

## Data Availability

All data generated or analyzed are included in the article. Other relevant data may be requested through the corresponding author.

## Abbreviations

BFAR-RMaTDeC: Bureau of Fisheries and Aquatic Resources - Regional Mariculture Technodermo Center)
bp: base pair
DA-BFAR: Department of Agriculture’s Bureau of Fisheries and Aquatic Resources
dNTPs: deoxyribonucleotide triphosphate
ELISA: enzyme-linked immunosorbent assay
EMJH: Ellinghausen, McCullough, Johnson and Harris
*flaB*: flagellin B
IgG: immunoglobulin G
IgM: immunoglobulin M
LAT: Latex Agglutination Test
MAT: Microscopic Agglutination Test
MgCl_2_: magnesium chloride
NCBI: National Center for Biotechnology Information
NE: northeast
PCR: Polymerase Chain Reaction
PMMSN: Philippine Marine Mammal Stranding Network
rDNA: ribosomal DNA
rRNA: ribosomal RNA
SW: southwest
TAE: Tris-acetate-EDTA
*Taq*: *Thermus aquaticus*
UV: Ultraviolet
